# Impact of thermal seed treatment on spermosphere microbiome, metabolome and viability of winter wheat

**DOI:** 10.1038/s41598-024-78575-0

**Published:** 2024-11-08

**Authors:** Maria E. Karlsson, Gustaf Forsberg, Anna Karin Rosberg, Christian Thaning, Beatrix Alsanius

**Affiliations:** 1 Dept of Biosystems and Technology, Microbial Horticulture Unit, Swedish University of Agricultural Sciences, PO Box 190, Lomma, 23244 Sweden; 2 Lantmännen BioAgri AB, Fågelbacksvägen 3, Uppsala, 75651 Sweden

**Keywords:** Winter wheat, Thermal treatment, Seed vigour, Metbolomics, Metagenomics, Biological techniques, Microbiology, Molecular biology, Plant sciences

## Abstract

Thermal seed treatment can be used as an alternative method to prevent infection by seed-borne diseases, but exposure duration and temperature during thermal treatment are important to maintain high seed viability and emergence whilst decreasing infection rate. A method for predicting suitable treatment parameters to maintain viability and eliminate seed-borne pathogens is therefore needed. Seeds of winter wheat were subjected to thermal treatment at four levels of intensity and pre-treatments with or without imbibition. Treatment impact was measured by metabolome analysis using LC-MS and GC-MS, analysis of spermosphere bacterial and fungal metagenomes using Illumina MiSeq, and detection of presence of *Fusarium* spp. and *Microdochium* spp. using ddPCR. The results showed that moderate treatment intensity reduced signs of infection and increased seedling emergence. In imbibed samples, myo-inositol concentration and myo-inositol: glucose ratio were positively correlated with treatment intensity, whereas concentrations of glucose and citric acid were negatively correlated. No correlations were found for non-imbibed samples. Imbibition had a large significant impact on microbial community composition of the wheat spermosphere. Imbibition of wheat seeds prior to thermal treatment altered wheat spermosphere microbiota. The concentration of myo-inositol, potentially in combination with glucose, could be a candidate predictor for suitable thermal treatment intensity of wheat seeds.

**Supplementary Information:**

The online version contains supplementary material available at 10.1038/s41598-024-78575-0.

## Introduction

Crop production is dependent on vigorous and healthy seeds that can withstand biotic and abiotic stress factors to produce a resilient crop. In order to reduce crop losses during emergence and control seed-borne diseases, commercial seeds are commonly treated with chemical-based fungicides. Thermal seed treatment reduces the use of pesticides and has been applied in different ways for many decades^1,2^. In thermal treatment, the balance between temperature, humidity and length of exposure is crucial for controlling pathogens, while still maintaining seed viability^3^. The most important measure of seed quality is germination, which refers to the ability of seed to germinate and produce a primary root (radicle). A more demanding measure of seed quality is seed emergence, which is the key characteristic of seed in creating strong and viable seedlings that can emerge quickly through the soil under stressful conditions. Emergence rate is thus of great value as an indicator of good establishment of seed in the field.

Seed germination and development of seedlings are crucial steps in the life cycle of plants, but the first symptoms of diseases such as *Septoria tritici* blotch and *Fusarium* head blight, which lead to reduced grain yield, can often be seen on the coleoptile soon after emergence. The causal agent in *Septoria tritici* blotch, a serious pathogen in wheat, is *Mycosphaerella graminicola*, (asexual stage: *Septoria tritici)*. In winter wheat, *Septoria tritici* infection reduces overwintering ability. Degree of leaf damage and seed infection both vary widely between years, depending on weather conditions. Infection of only the lower leaves causes little damage, whereas infection of flag leaves and axes can reduce yield by 20–30%^4^. *Fusarium* head blight is a pre-harvest disease, but if conditions are right and the harvested grains are not fully dry, this pathogen can also grow post-harvest^5^. The most frequently isolated *Fusarium* spp. on wheat are *F. culmorum*,* F. graminearum* and *F. avenaceum*. *Fusarium*-infected seeds are less vigorous and can contain mycotoxins, especially deoxynivalenol (DON), causing food and feed contamination^6^.

The seed-associated microbiota influences seed viability and germination^7^ and is therefore related to plant fitness^8^. This interaction contributes to spermosphere microbial community composition, which is transferred during plant development^9^. The microbiota colonizes seeds both endophytically and epiphytically, and it is unclear whether the microbes that end up on seedlings originate from endophytic or epiphytic microbiota^10^. From an ecological perspective, the seed microbiota represents an endpoint for the microbial community within the seed and a starting point for microbial community structure on the seedling^8^.

The metabolome, or metabolic profile, refers to the range of metabolites of a species present at a given time, or in a given situation. The metabolome determines how well the metabolism of an organism can adapt to various conditions and mediate the communication between genome and phenotype of the organism. It can therefore be a potential tool in determining biochemical responses of a host to different abiotic and biotic stressors, as demonstrated in various areas of application and organisms^11,12^. In wheat, metabolome studies have been used to identify metabolic pathways activated following infection with plant pathogens^13,14^ and to responses for example drought stress^15^.

The aims of the present study were: to determine whether thermal treatment intensity affects the metabolome and microbial community of winter wheat seeds. We also evaluated the effect of thermal seed treatment on seed viability, signs of fungal infection and presence of viable *Fusarium* spp. and *Microdochium* spp. Finally we investigated whether some key metabolites and key organisms can predict the viability of winter wheat seeds.

## Materials and methods

All methods used in this study comply with relevant institutional, national and international guidelines and legislation.

### Seed and seed treatment

Numerous winter wheat (*Triticum aestivum*) seed grown at commercial wheat production sites in Sweden with high prevalence of mixed fungal infection were used in this study. Thermal treatment with ThermoSeed^®^ processing equipment at different heat intensities 0, 3, 5, 6 and 7 on the ThermoSeed scale, corresponding to 50–90℃) was performed at ThermoSeed Global AB (Uppsala, Sweden). The samples were divided into two groups, one of which was imbibed in oxygenated water for 48 h, while the other group was not imbibed. Three different sample sets were used: (i) a small set of 36 samples (6 replicates per treatment) of milled winter wheat was used in all analyses (viability, metabolomics, metagenomics); (ii) a large set of 170 samples of whole seeds was used for molecular analysis (metagenomics, quantification of fungi with ddPCR); and (iii) validation of viability and metabolomics analyses was performed using only imbibed samples (*n* = 85, 17 replicates per treatment).

### Microbial analysis

For each sample, 100 mg milled winter wheat seeds were used (in total 36 samples). For whole winter wheat seeds (170 samples), 10 g of seeds were placed in 50-mL Falcon tubes together with 10 mL PBS (Phosphate buffered saline). The tubes were agitated on a shaker for 4 h, transferred to a stomacher bag and macerated (Smasher; bioMérieux, Inc., 100 Rodolphe Street, Durham, NC 27712, U.S.A.) for 30 s at normal speed. The liquid was poured back into the tube followed by centrifugation at 5000 rpm for 10 min. The supernatant was discarded and pellet was suspended in 750 µL RNA/DNA shield (Zymo Research). DNA and RNA were extracted with a Zymobiomics DNA/RNA miniprep Kit (Zymo Research) according to the manufacturer’s protocol.

### ddPCR (droplet digital PCR)

*Fusarium avenaceum*,* F. culmorum*,* F. graminaceum*,* Microdochium nivale* and *M. nivale majus* were quantified using the automated QX200TM Droplet Digital™ PCR system (Bio-Rad, Hercules, CA, USA). A reaction mix composed of 10 µL QX200 EvaGreen Digital PCR Supermix, 0.5 µL each of forward and reverse species-specific primers (Supplementary Table S1), 7 µL DNase/RNase free MilliQ water and 2 µL cDNA sample was prepared (final volume 20 µL). Samples were placed in the automated droplet generator. The plate containing droplets was sealed with pierceable aluminium foil using a PX1 PCR plate sealer (Bio-Rad, Hercules, CA, USA) set to 180 ℃ for 5 s. The plate was then moved to a Touch Thermal Cycler (Bio-Rad, Hercules, CA, USA) and run with the following thermal conditions: Enzyme activation 95℃ for 5 min following by 40 cycles of denaturation at 95 ℃ for 30 s, annealing and extension for 1 min with the temperature specific for the primer used. The procedure ended with signal stabilisation at 4 ℃ for 5 min and 90 ℃ for 5 min and infinite hold at 4 ℃. After thermal cycling, the plate was placed in a QX droplet reader (Bio-Rad, Hercules, CA, USA). QuantaSoft™ software was used to run the instrument and analyse the data.

### Illumina sequencing metagenomics

Microbial community composition of the 36 milled winter wheat seeds and the 170 samples of whole winter wheat seeds was analysed using llumina MiSeq, with 300 bp paired end reads, at LGC Genomics GmbH (Berlin, Germany). Bacterial communities were assessed by targeting the 16S ribosomal gene using the primer combination forward primer 799F (5′-AACMGGATTAGATACCCKG-3′)^16^ and reverse primer 1115R (5′-AGGGTTGCGCTCGTTRC-3′)^17^. To assess fungal communities, the forward primer ITS1F_Kyo2 (5′-TAGAGGAAGTAAAAGTCGTAA-3′)^18^ and the reverse primer (5′-TTCAAAGATTCGATGATTCAG-3′)^19^ were used to target the ITS region.

Data obtained in Illumina sequencing were analysed by the bioinformatics service at LGC Genomics GmbH (Berlin, Germany) which also performed quality control on all data. In brief, the Illumina bcl2fastq v2.20 software was used to demultiplex all libraries for each sequencing lane. The barcode sequence was clipped from the sequence after sorting and reads with missing barcodes, one-sided barcodes or conflicting barcode pairs were discarded, as were reads with final length < 100 bases. Mothur 1.35.1 was used for community diversity analysis. Clustering of operational taxonomic units (OTUs) of the fungal community was carried out at 97% identity level, with cluster representative sequence to the most abundant sequence, instead of the default representative sequence (longest sequence). The prokaryotic sequences were aligned against the 16 S Mothur-Silva SEED r 119 reference alignment. The fastTree v2.1.7 method was used to generate *de novo* phylogenetic trees for both fungal and bacterial communities.

### Metabolome analysis

Metabolome analysis was performed at the Swedish Metabolomic Centre (SMC) in Umeå. Seeds were milled twice and sent to SMC, where samples were freeze-dried to compensate for any difference in water content between the treatments before metabolite extraction. Metabolite extraction was performed according to (Vancov and Keen, 2009)^19^. In detail, 1000 µL extraction buffer (20/20/60 v/v chloroform: water: methanol) including internal standards were added to 2–3 mg freeze-dried material. The sample was shaken with a tungsten bead in a mixer mill at 30 Hz for 3 min. After shaking, the bead was removed and the sample was centrifuged at 4 ℃, 14 000 rpm, for 10 min. The supernatant was transferred to micro-vials and evaporated to dryness in a speed-vac concentrator. A 200 µL sample was used for liquid chromatography-mass spectrometry (LC-MS) analysis and a 50 µL sample for gas chromatography-mass spectrometry (GC-MS) analysis. Solvents were evaporated and the samples were stored at -80 ℃ until analysis. A small aliquot of the remaining supernatant was pooled and used to create quality control (QC) samples. LCMS analysis was run on the QC samples for identification purposes.

The samples were analysed in batches, according to a randomised run order, in both GC-MS and LC-MS. For GC-MS analysis, 0.5 µL derivatised sample was injected in splitless mode by a L-PAL3 autosampler (CTC Analytics AG, Switzerland) into an Agilent 7890B gas chromatograph equipped with a 10 m x 0.18 mm fused silica capillary column with a chemically bonded 0.18 μm Rxi-5 Sil MS stationary phase (Restek Corporation, USA). The injector temperature was 270 ℃, the purge flow rate was 20 mL min^− 1^ and the purge was turned on after 1 min. The gas flow rate through the column was 1 mL min^− 1^. The column temperature was held at 70 ℃ for 2 min, then increased by 40 ℃ min^− 1^ to 320 ℃, and held there for 2 min. The column effluent was introduced into the ion source of a Pegasus BT gas chromatograph-time-of-flight mass spectrometer (GC-TOFMS) (Leco Corp., St Joseph, MI, USA). The transfer line temperature was 250 °C and the ion source temperature was 200 ℃. Ions were generated by a 70 eV electron beam at an ionisation current of 2.0 mA, and 30 spectra s^− 1^ were recorded in the mass range m/z 50–800. The acceleration voltage was turned on after a solvent delay of 150 s. The detector voltage was 1800–2300 V.

Before LC-MS analysis, the sample was re-suspended in 10 µL methanol and 10 µL water. Each batch of samples was first analysed in positive mode. After all samples within a batch had been analysed, the instrument was switched to negative mode and a second injection of each sample was performed.

Chromatographic separation was performed on an Agilent 1290 Infinity UHPLC-system (Agilent Technologies, Waldbronn, Germany). Aliquots of 2 µL of each sample were injected onto an Acquity UPLC HSS T3, 2.1 × 50 mm, 1.8 μm C18 column in combination with a 2.1 mm x 5 mm, 1.8 μm VanGuard pre-column (Waters Corporation, Milford, MA, USA) held at 40 ℃. The gradient elution buffers were A (H2O, 0.1% formic acid) and B (75/25 acetonitrile:2-propanol, 0.1% formic acid), and the flow-rate was 0.5 mL min^− 1^. The compounds were eluted with a linear gradient consisting of 0.1–10% B over 2 min, after which B was increased to 99% over 5 min, held there for 2 min and decreased to 0.1% for 0.3 min. The flow rate was increased to 0.8 mL min^− 1^ for 0.5 min. These conditions were held for 0.9 min, after which the flow-rate was reduced to 0.5 mL min^− 1^ for 0.1 min before the next injection.

Compounds were detected with an Agilent 6550 Q-TOF mass spectrometer equipped with a jet stream electrospray ion source operating in positive or negative ion mode. The settings were kept identical between the modes, with exception of the capillary voltage. A reference interface was connected for accurate mass measurements. The reference ions purine (4 µM) and HP-0921 (Hexakis(1 H, 1 H, 3 H-tetrafluoropropoxy)phosphazine) (1 µM) were infused directly into the MS at a flow rate of 0.05 mL min^− 1^ for internal calibration. The monitored ions were purine m/z 121.05 and m/z 119.03632 for positive mode; and HP-0921 m/z 922.0098 and m/z 966.000725 for negative mode. The gas temperature was set to 150 ℃, the drying gas flow to 16 L min^− 1^ and the nebulizer pressure to 35 psig. The sheath gas temperature was set to 350 ℃ and the sheath gas flow to 11 L min^− 1^. Capillary voltage was set to 4000 V in positive ion mode and to 4000 V in negative ion mode. The nozzle voltage was 300 V. The fragmenter voltage was 380 V, the skimmer voltage 45 V and the OCT voltage 1 RF Vpp 750 V. The collision energy was set to 0 V. The m/z range was 70-1700, and data were collected in centroid mode with an acquisition rate of 4 scans s^− 1^ (1977 transients/spectrum).

### Viability analysis

Viability tests were performed as follows: 1 kg winter wheat seeds were cleaned in a sample cleaner (MLN model from Perten) to remove debris and dust to obtain uniform seed size. The sample was then divided and treated under the same conditions as described in the section ‘Seed and seed treatment’. After heat treatment, the seeds were sown in soil at a depth of 3 cm, with 50 seeds per replicate and three replicates per treatment, in transparent plastic pots (185 × 125 × 75 mm) with two holes at the bottom for easy and uniform watering. The pots were randomised on a tray and covered with lids to keep the soil from drying out. The tray was placed at 6 ℃ in darkness for 10 days (cold incubation) and then moved to a 20 ℃ chamber under three sets of polychromatic LED lights for 24 h, where pot lids were removed and the pots were watered from the bottom with 5 L tap water. After five days, each pot was evaluated for number of emerged seedlings out of 50 that had reached the soil surface. Seven days later, the top layer of the soil was removed to evaluate the infection status of each plant based on the appearance of the coleoptile. Coleoptiles showing any brown lesions were considered diseased, while white/green coleoptiles were considered healthy.

### Statistical analysis

All statistical analyses (ANOVA, Dunnett´s, PCA and PCoA) were performed in R studio (http://www.rstudio.com). In order to estimate changes in the microbial community in the different heat intensity treatments, Shannon index and Chao1 index were used to estimate alpha diversity using the package phyloseq^20^. Beta diversity was calculated using weighted UniFrac in the *distance* and *ordinate* function in the Phyloseq package. For the GC-MS data, all non-processed MS files from the metabolic analysis were exported from the ChromaTOF software in NetCDF format to MATLAB R2020a (Mathworks, Natick, MA, USA), where all data pre-treatment procedures, such as base-line correction, chromatogram alignment, data compression and Multivariate Curve Resolution were performed. The extracted mass spectra were identified by comparisons of their retention index and mass spectra with libraries^21^. Mass spectra and retention index comparison was performed using NIST MS 2.2 software. Annotation of mass spectra was based on reverse and forward searches in the library. The mass spectrum with the highest probability indicative of a metabolite and the retention index between the sample and library for the suggested metabolite was ± 5 (usually < 3). The deconvoluted “peak” was annotated as an identification of a metabolite. For the LC-MS data, all data processing was performed using the Agilent Masshunter Profinder version B.10.00 (Agilent Technologies Inc., Santa Clara, CA, USA). Data pre-processing was performed in an untargeted fashion, using the Batch Recursive Feature Extraction algorithm within Masshunter Profinder.

## Results

### Emergence and infection

The occurrence of infection was affected by the heat treatment, with infection rate decreasing with increasing intensity of heat treatment. The results from the small set of samples (*n* = 36) showed, at heat intensity 5, imbibed samples showed no significant difference in infection rate to the untreated control (Table 1). Thermal treatment at heat intensity 5 had a significant positive effect (*p* < 0.001) on emergence in seedlings, whereas thermal treatment at heat intensity 7 had a significant negative impact (*p* = 0.001) (Table 1). Imbibition did not alter seedling emergence.

**Fig. 1:**
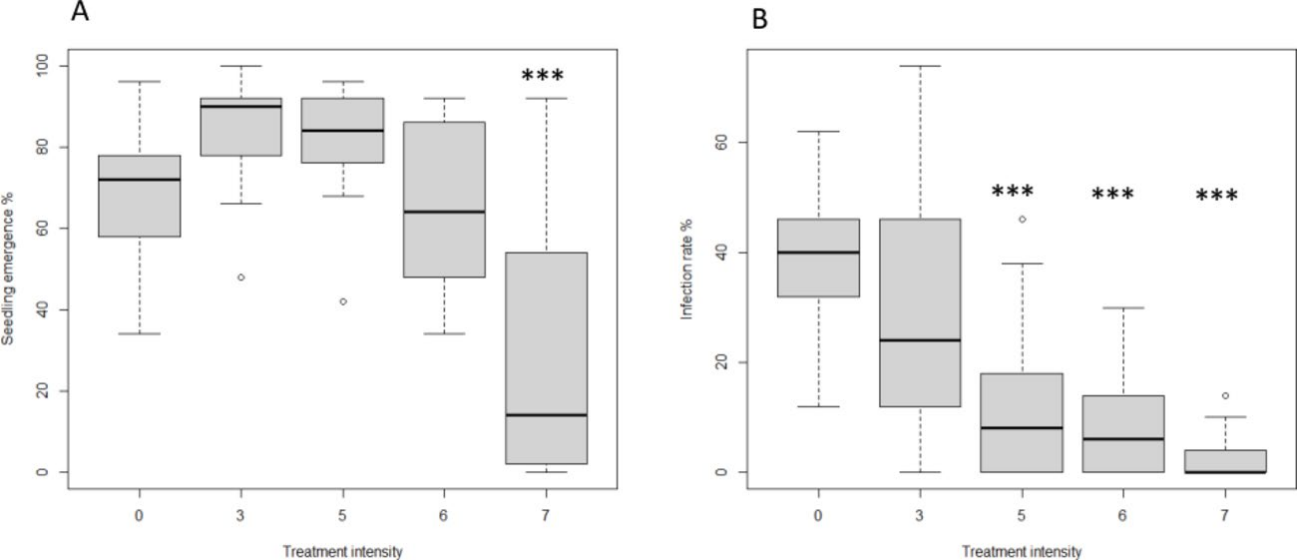
Boxplots showing effect of heat treatment intensity on (**A**) percentage seedling emergence of winter wheat and (**B**) percentage infection rate. Results shown are only for imbibed samples and five different heat treatment intensities (*n* = 85). Asterisks above bars indicates significant difference (*p* < 0.05).

The results from the emergence test on the large sample set with only imbibed samples (*n* = 85) also showed a significantly negative effect of high heat treatment on seedling emergence (*p* = 0.003, Fig. 1A). Heat treatment with the intensity 5 did not affect seedling emergence negatively. Regarding the infection rate, there was a negative significant effect of heat treatment intensity (p = < 0.0001, Fig. 1B). The infection rate explained 22% of the variance in seedling emergence (*p* = 0.047).

**Table 1:** Effect of heat treatment intensity on seedling emergence and infection rate of winter wheat seedlings (n = 36). Results of ANOVA and Dunnet’s test against control samples (no heat treatment and non-imbibed).

	Diff	Lower 95%CI	Upper 95%CI	P value
Emergence				
Treatment				
Imbibed 7—Control	− 20.383	− 28.688	− 12.078	**0.0001**
Imbibed 0—Control	− 2.716	− 11.021	5,588	0.85
Imbibed 5—Control	13.7	5.395	22.005	**0.0001**
Not imbibed 7—Control	− 21.833	− 30.138	− 13.528	**0.0001**
Not imbibed 5—Contrl	15.616	7.311	23.921	**0.0001**
Infection				
Treatment				
Imbibed 7—Control	− 13.850	− 19.575	− 8.125	** < 0.0001**
Imbibed 0—Control	1.817	− 3.908	7.542	0.87
Imbibed 5—Control	− 1.183	− 6.908	4.542	0.97
Not imbibed 7—Control	− 13.750	− 19.475	− 8.024	** < 0.0001**
Not imbibed 5—Control	− 7.033	− 12.758	− 1.307	**0.01**

### Metabolomics

Metabolome patterns clearly differed between imbibed and non-imbibed samples. However, untargeted LC-MS metabolomics analysis were not able to predict the required intensity of heat treatment compared with untargeted GC-MS metabolomics analysis.​.

**Fig. 2:**
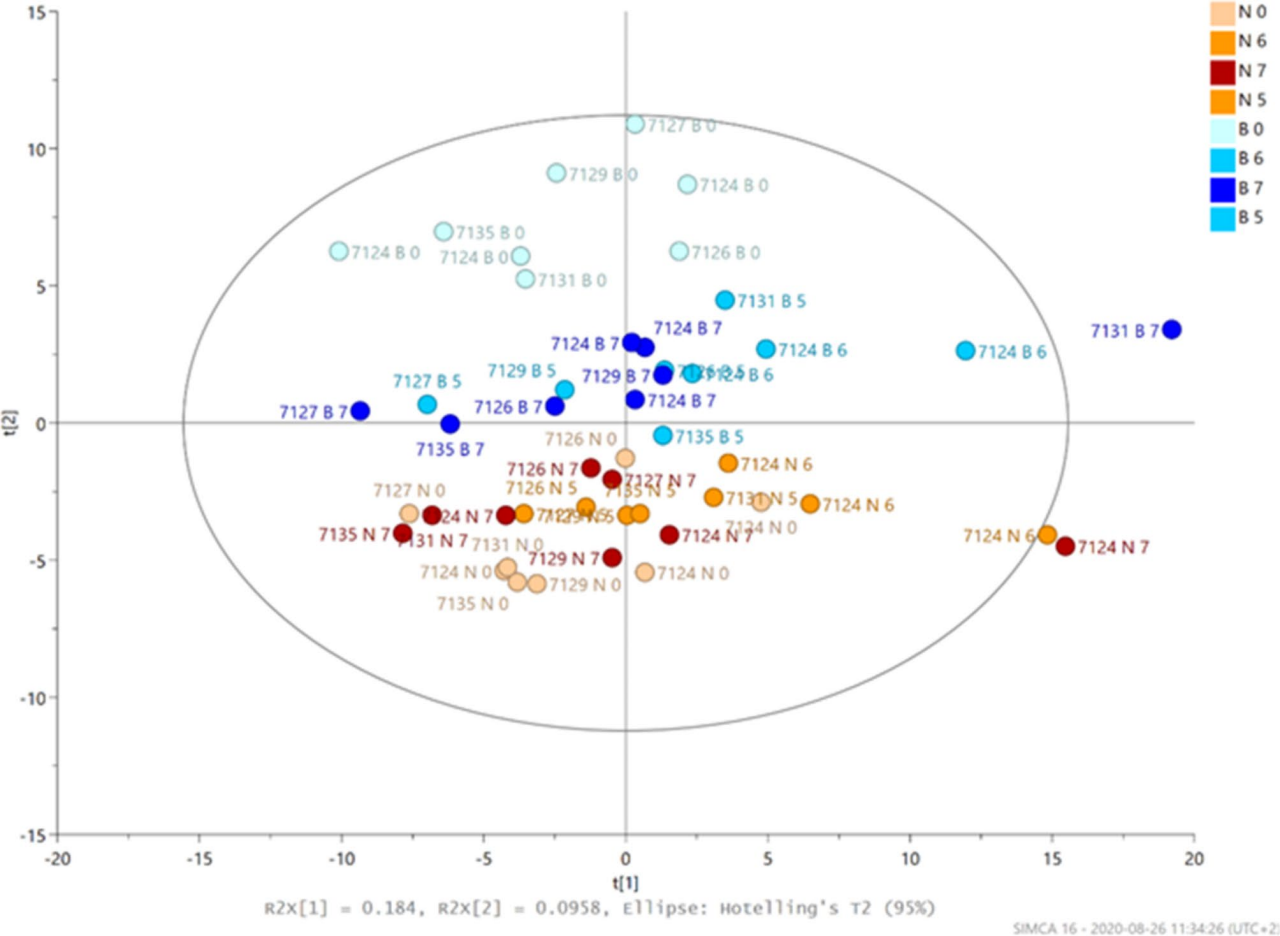
Principal component analysis (PCA) plots showing differences between imbibed (B-samples, blue dots) and non-imbibed (N-samples, red dots) exposed to no heat treatment or to moderate (5 and 6), and high (7) heat treatment intensities (*n* = 36).

**Fig. 3:**
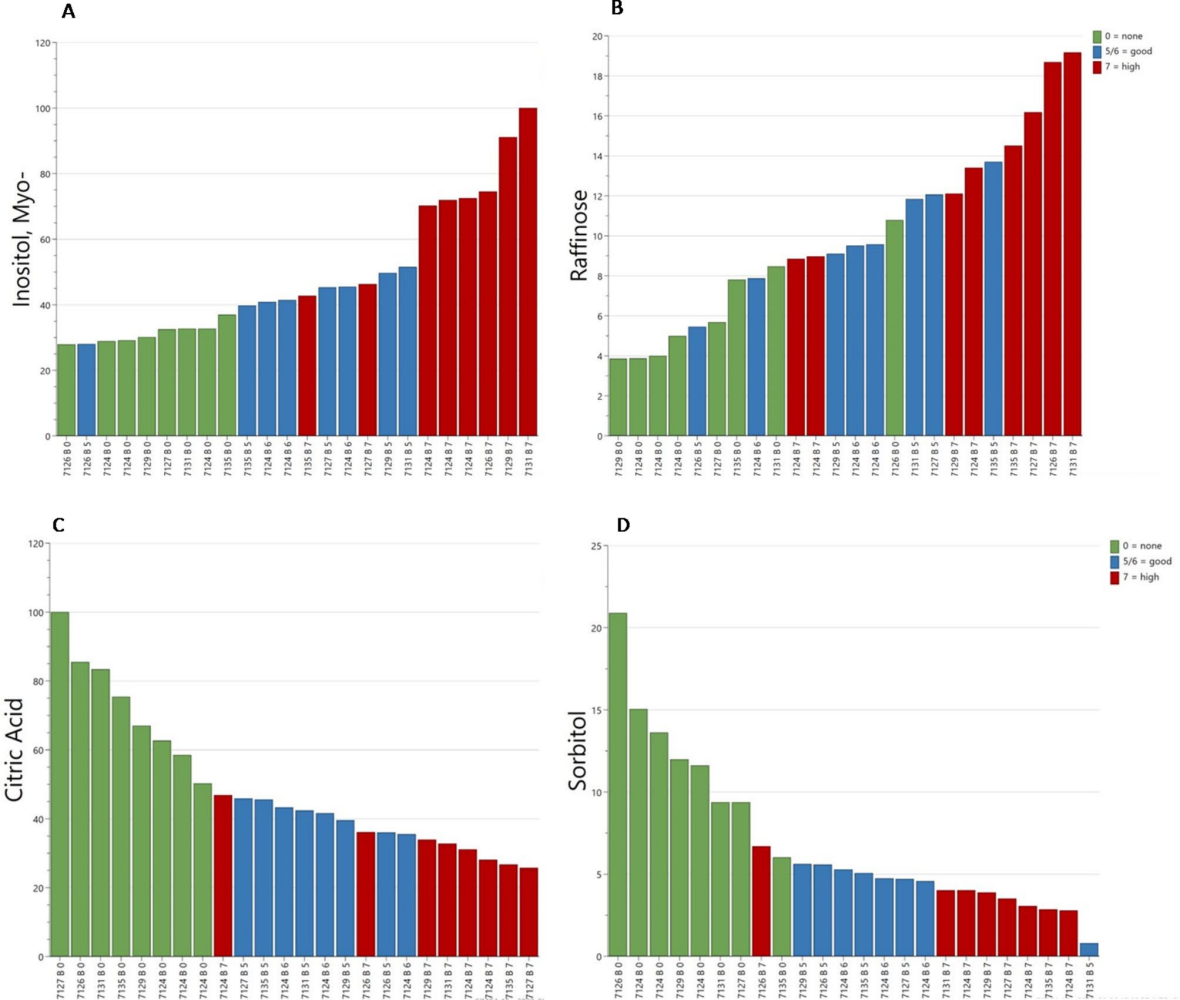
Annotated metabolites increase in concentration due to intensity of treatment​ (**A**) Mya-Inositol (**B**) Raffinose. Annotated metabolites reduce in concentration due to intensity of treatment​ (**C**) Citric Acid (**D**) Sorbitol.

GC-MS analysis showed a clear difference between imbibed and non-imbibed samples, where the imbibed seeds were clearly separated in PCA plots by the different heat treatment intensities (Fig. 2). It was possible to predict the required intensity of heat treatment based on the metabolites myo-inositol, citric acid, sorbitol and raffinose. The concentrations of myo-inositol and raffinose increased with increased thermal treatment intensity, while the concentrations of citric acid and sorbitol decreased (Fig. 3A-D).

**Fig. 4:**
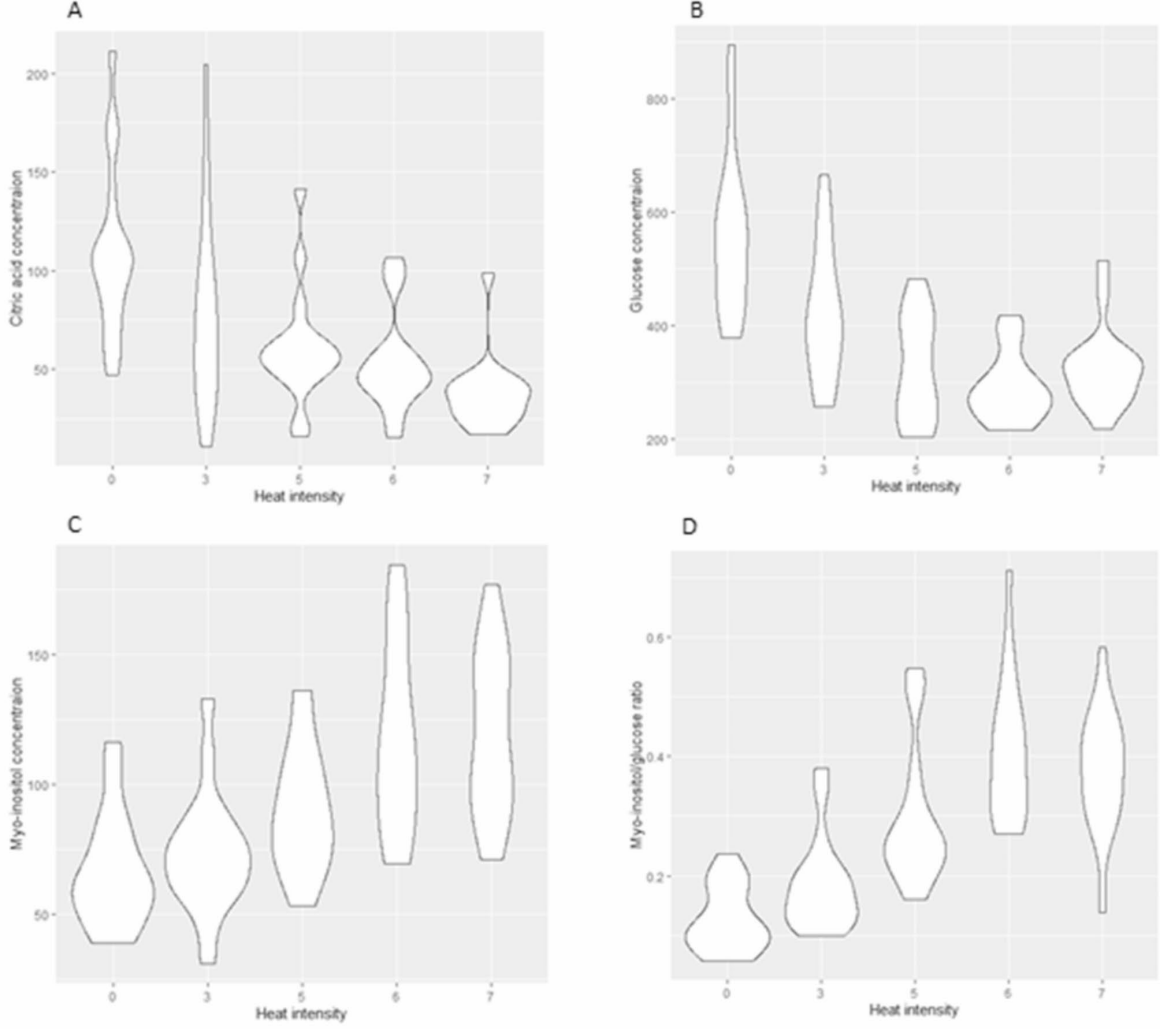
Violin plot showing the effect of different heat intensities in the imbibed samples (*n* = 85) on the concentration of (**A**) citric acid, (**B**) glucose and (**C**) myo-inositol and (**D**) myo-inositol: glucose ratio.

In order to validate the results from the first run of metabolomics, a second run was performed with only imbibed samples. The results confirmed that the concentrations of citric acid (t=-8.35, df = 83, *p* < 0.0001; Fig. 4A) and glucose (t=-6.54, df = 83, *p* < 0.0001; Fig. 4B) were negatively correlated with heat treatment. Myo-inositol concentration was positively correlated with heat treatment (t = 6.48, df = 83, *p* < 0.0001; Fig. 4C), and myo-inositol: glucose ratio was positively correlated with heat treatment intensity (t = 7.53, df = 83, *p* < 0.0001; Fig. 4D).

### Interactions between metabolome, seedling emergence and infection

**Fig. 5:**
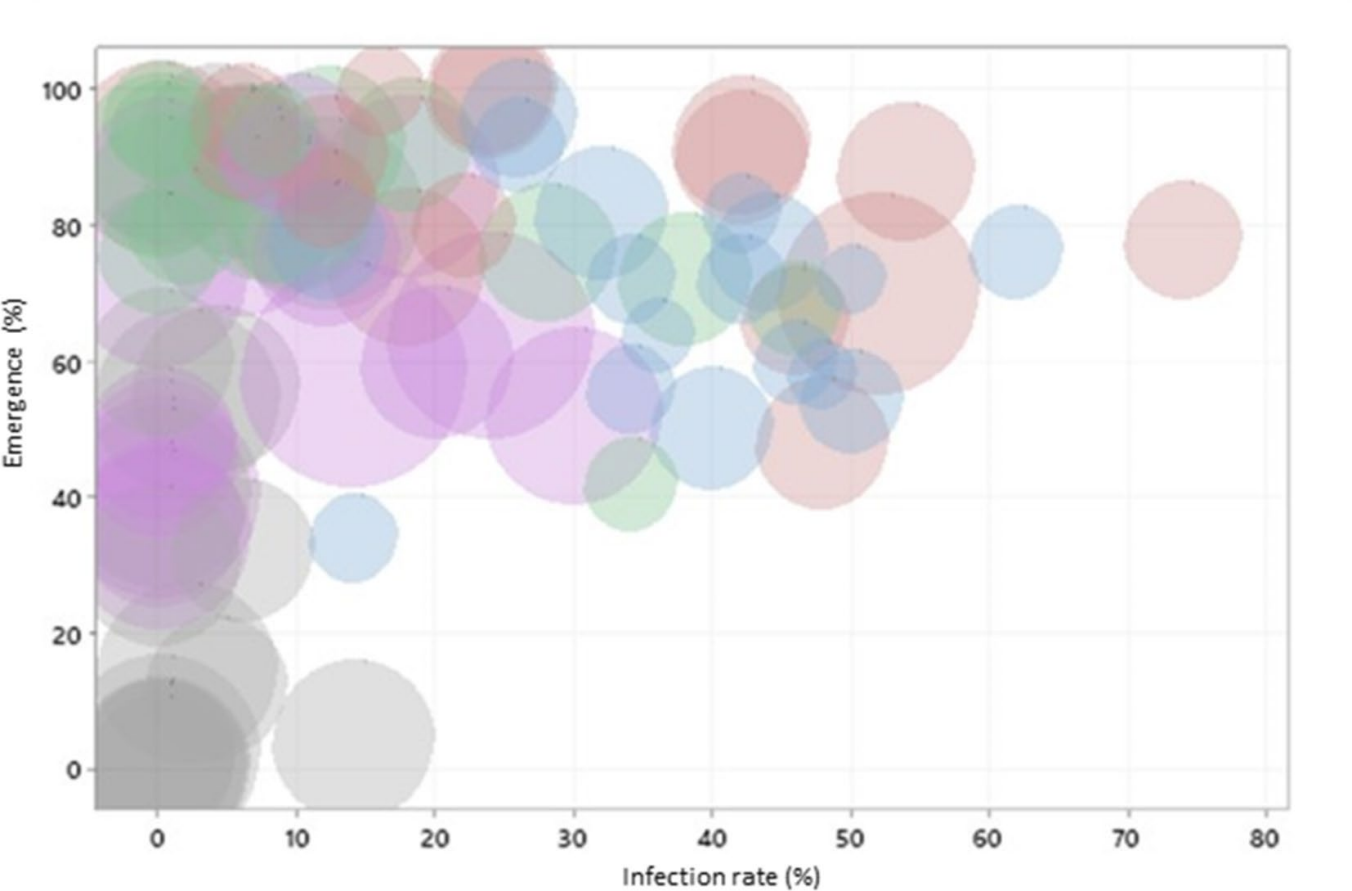
Concentration of myo-inositol in response to the interaction between seedling emergence (%) and infection rate (%) in winter wheat exposed to different heat treatment intensities (blue: no heat treatment, red: heat treatment intensity 3, green: heat treatment intensity 5, purple: heat treatment intensity 6, grey: heat treatment intensity 7). Myo-inositol concentration indicated by bubble size.

Low concentrations of myo-inositol were correlated with high seedling emergence (R^2^=-31.0%; *p* = 0.004) and also with high disease incidence (R^2^=-55.5%; *p* < 0.001). Disease incidence was positively correlated with seed glucose concentration (R^2^ = 42.2%; *p* < 0.001). In addition, there was a tendency for a direct interaction between seedling emergence and glucose concentration, although Pearson correlation was not statistically significant for this. Myo-inositol: glucose ratio showed the strongest interaction for both emergence (R^2^=-30.4%; *p* = 0.005) and disease incidence (R^2^=-57.8%; *p* < 0.001) (Fig. 5 ).

### Metagenomics

The microbial community identified included both endophytic and epiphytic microorganisms. No effect of heat treatment on relative abundance of either bacteria or fungi was observed. However, there was a clear difference in bacterial relative abundance when seeds were imbibed, or not, before being milled. At phylum level, Proteobacteria was the dominant phylum in both imbibed and non-imbibed samples (relative abundance > 2%), but imbibed samples had lower relative abundance of Actinobacteria than non-imbibed samples (Supplementary Fig. S1A). The difference between imbibed and non-imbibed samples was still present on bacterial family and genus level. On family level, imbibed samples were dominated by *Oxalobacteraceae*, but in non-imbibed samples the dominant taxon was *Enterobacteriaceae* (Supplementary Fig. S2A). At genus level, there was a shift in relative abundance of *Pantoea*, *Pseudomonas* and *Curtobacterium*, with lower abundance in imbibed samples (Supplementary Fig. S3A).

The fungal community was dominated by *Ascomycota* in all treatments (Supplementary Fig. S1B). The family *Xylariales* dominated in all treatments, but the relative abundance of *Pleosporales* was lower in imbibed samples (Supplementary Fig. S2B). At genus level, *Monographella* was dominant in all treatments and relative abundance of *Xenobotryosphaeria* and *Ascochyta* was higher in non-imbibed samples (Supplementary Fig. S3B). At species level, the dominant taxon was *Monographella nivalis* in all treatments, with a decrease in relative abundance of *Xenobotryosphaeria calmagrostidis and Ascomycota* sp. in the imbibed samples (Supplementary Fig. S4).

**Fig. 6:**
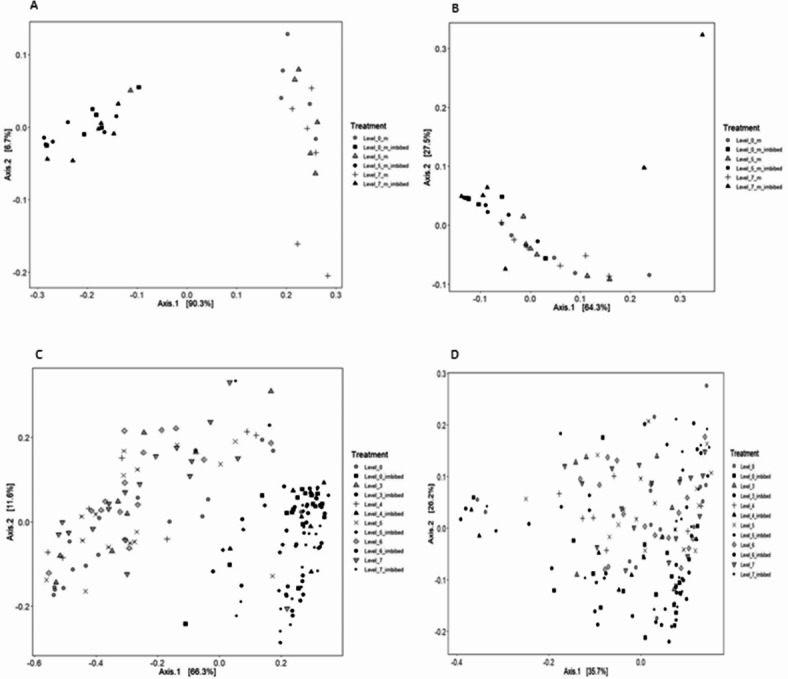
Principal coordinate analysis (PCoA) plots of beta diversity based on weighted UniFrac distances for (**A**) 16 S rRNA bacterial community on milled winter wheat seeds, (**B**) ITS fungal community on milled winter wheat seeds, (**C**) 16 S rRNA bacterial community on winter wheat seed coats and (**D**) ITS fungal community on winter wheat seed coats. Black = imbibed, grey = non-imbibed.

Alpha diversity assessed with the Shannon and Chao1 indices grouped the samples by heat treatment intensity and whether the samples had been imbibed or not. The Shannon diversity index for bacteria showed a significant difference (*p* < 0.01) between the control and imbibed seeds exposed to moderate or high heat treatment intensities (Table 2). Community richness (Chao1) significantly increased (*p* < 0.01) in imbibed seeds in all heat treatments (Table 2). The Shannon diversity index for fungi showed a significant difference (*p* < 0.01) between the control and the imbibed samples in all three heat treatments (Table 2). However, there was no significant impact on fungal community richness (Table 2). The beta diversity metrics for bacterial and fungal communities showed no clear distinction between heat treatments, but in PCoA plots bacterial community clustered with respect to imbibition treatment, which was not seen for the fungal community (Fig. 6A and B).

**Table 2:** Impact of heat treatment intensity on alpha diversity (Shannon and Chao1) of bacteria and fungi (n = 36). Results of ANOVA and Dunnett’s test against control samples (no heat treatment and non-imbibed).

Treatment	Difference	Lower 95% CI	Upper 95% CI	P value
Shannon (Bacteria)				
Imbibed 7—Control	− 0.840	− 1.564	− 0.115	**0.01**
Imbibed 0—Control	− 0.614	− 1.340	0.111	0.12
Imbibed 5—Control	− 0.984	− 1.708	− 0.259	**0.005**
Not imbibed 7—Control	− 0.713	− 1.438	0.012	0.06
Not imbibed 5—Control	− 0.259	− 0.984	0.464	0.81
Chao 1 (Bacteria)				
Imbibed 7—Control	18.608	1.063	36.152	**0.03**
Imbibed 0—Control	37.534	19.990	55.079	** < 0.0001**
Imbibed 5—Control	41.116	23.571	55.661	** < 0.0001**
Not imbibed 7—Control	− 11.151	− 28.696	6.392	0.32
Not imbibed 5—Control	5.918	− 11.626	23.462	0.83
Shannon (Fungi)				
Imbibed 7—Control	− 0.996	− 1.329	− 0.664	** < 0.0001**
Imbibed 0—Control	− 0.363	− 0.696	− 0.030	**0.03**
Imbibed 5—Control	− 0.881	− 1.213	− 0.548	** < 0.0001**
Not imbibed 7—Control	− 0.566	− 0.389	0.726	0.98
Not imbibed 5—Control	− 0.002	− 0.335	0.331	0.98
Chao 1 (Fungi)				
Imbibed 7—Control	2.452	− 15.651	20.736	0.99
Imbibed 0—Control	− 1.869	− 20.064	16.324	0.99
Imbibed 5—Control	5.794	− 12.40	23.988	0.86
Not imbibed 7—Control	2.653	− 15.541	20.847	0.99
Not imbibed 5—Control	6.604	− 11.59	24.798	0.79

The epiphytic microbial community on whole-wheat seeds showed the same pattern as seen for the milled samples, i.e. heat treatment did not affect microbial community structure. At phylum level, three phyla were observed. Proteobacteria was the dominant phylum in both imbibed and non- imbibed samples, followed by Firmicutes and Actinobacteria, which were reduced in abundance in the imbibed samples (Supplementary Fig. S5A). At family level, there were also differences between imbibed and non-imbibed samples of whole wheat seeds, e.g. *Enterobacteriaceae* was present in higher relative abundance in non-imbibed than imbibed samples (Supplementary Fig. S6A). At genus level, the dominant genera were *Pseudomonas*,* Microbacterium* and *Actinebacter*in in imbibed samples, *Pantoea* and *Curtobacterium* in non-imbibed samples (Supplementary Fig. S7A). Relative fungal abundance at phylum level showed no difference between the heat treatment intensities or imbibition/no imbibition, with the dominant phylum in all treatments being Ascomycota (Supplementary Fig S5B). At family level, heat treatment intensity affected the relative abundance of *Davidiellaceae* on whole wheat seeds, which increased with increasing heat treatment intensity, and Pleosporales was the dominant family (Supplementary Fig. S6B). The dominant genera in all treatments were *Xenobotryosphaeria*, *Monographella* and *Davidella* (Figure S7B in SI). At species level, the fungal community contained several plant pathogens, including *Parastagnospora pseudovitensis*,* Monographella nivalis* and *Fusarium poae* (Supplementary Fig. S8).

There was no impact of heat treatment intensity, or imbibition, on the alpha diversity (Shannon and Chao 1 indices) of either bacterial or fungal communities on whole winter wheat seeds. Beta diversity of bacteria did not separate between heat treatment intensities, but samples clustered with respect to imbibition treatment. Beta diversity of the fungal community did not discriminate between heat treatment intensities or imbibition treatment (Fig. 6C and D).

### ddPCR

ddPCR was used to estimate the prevalence of *Fusarium* spp. and *Microdochium* spp. on winter wheat seeds after heat treatment. Despite heat treatment, these pathogens were still present on the seeds. There were no differences between imbibed and non-imbibed samples (Table 3).

**Table 3:** Results from ddPCR, expressed as mean number of copies/µL detected on winter wheat seeds. NI = non-imbibed, I = imbibed (n = 170).

Treatment	*F. avenaceum* No copies/µl	*F. culmorum* No copies/µl	*F. graminaceum* No copies/µl	*M. nivale* No copies/µl	*M. nivale majus* No copies/µl
NI 0	7.98	47.71	13.84	432	2.76
NI 3	8.05	36.38	14.15	135.47	1.21
NI 5	6.00	27.98	13.44	100.55	3.06
NI 6	7.62	27.98	21.06	306	1.12
NI 7	4.41	34.16	8.22	139.86	0.89
I 0	5.88	54.77	8.55	29.9	1.26
I 3	15.04	86.97	20.52	30.49	1.95
I 5	6.77	51.20	8.24	8.89	2.60
I 6	12.08	53.31	22.00	220.64	1.63
I 7	7.96	58.17	9.00	5.65	1.47

## Discussion

Thermal treatment of seeds can reduce the prevalence of pathogen attack and enhance seed emergence, as demonstrated by the bioassays performed in the present study. Heat treatment intensity modified the concentration of some sugars and organic acids, including myo-inositol, which is an important metabolite for plant development and growth^22^. It is also important for auxin storage, cell wall biogenesis and synthesis of phytic acid and oligosaccharides such as raffinose^23^. Oligosaccharides of the raffinose family are considered to play an important role in seed vigour and to regulate seed germination^24–26^. Raffinose is known to impart protection against desiccation of seeds^27^, but has also been detected in the leaves of heat-stressed plants (*Arabidopsis*)^25^. This could explain the increasing raffinose concentration in seed samples with increasing heat treatment intensity in this study. Additionally, the concentration of myo-inositol increased with the infection rate, which has been demonstrated in another study where metabolic profiles changed in wheat grains after infection with *Tilletia controversa* Kühn^13^. Glucose is very important for early seed development, where it is used in metabolic pathways for example in biosynthesis of myo-inositol^28,29^. The ratio between myo-inositol and glucose partly explained the variation in infection rate after heat treatment. The occurrence of endophytic pathogens might be one factor explaining the remaining variance. Heat treatment using ThermoSeed^®^ has been shown to affect epiphytic organisms, whereas endophytic organisms remain viable^30^. Based on cDNA analysis in the present study, *Fusarium* spp. and *Microdochium* spp. still prevailed post-treatment and reads of *Microdochium nivale* were high compared with the other fungi. However, while pathogens were still present according to molecular analysis with ddPCR, the bioassay results indicated that heat treatment may have had a negative effect on their pathogenicity.

The results obtained in this study indicate that myo-inositol: glucose ratio may be a suitable candidate for determination of disease incidence and seedling emergence after heat treatment, and can thus act as predictor of the heat treatment intensity required to control seed disease while maintaining emergence in individual lots of winter wheat seeds. However, the correlation coefficients were not always strong. Thus, to validate and further verify myo-inositol: glucose ratio as a predictor, the window for heat treatment intensities needs to be narrowed to 4-6.5 on the ThermoSeed^®^ scale, which corresponds to 50–70 ℃. Temperature and time intervals are important factors in seed emergence^3^.

No single group of microorganisms were linked to heat treatment intensity in metagenomics based on DNA extracted from seed coats and milled seeds of winter wheat (i.e. DNA from both dead and living microorganisms). The treatment with imbibition clearly changed the bacterial community composition where the relative abundance of the family *Entreobacteriaceae* increased in the not imbibed samples and there was a decrease of the relative abundance of *Oxalobacteraceae*. The opposite was noted in the imbibed samples. Microbial community diversity and richness was not affected by the concentration of any metabolite. However, a certain carefulness should be considered when looking for associations between metabolites and data from metagenomics since metabolomics profiles can be shared between plants and microorganisms. The relationship between metabolites and microorganisms is complex. They not only can be used by a microorganism as a source of nutrients or as an indicator for stress, they can also be secreted by the organism itself. Both plant and microbial metabolites could be key drivers in the distribution and survival of different microbial communities. To detect a significant change in community composition, the impact of any treatment needs to exceed the load of DNA from dead organisms. Thus, to identify a specific organism or group of organisms predicting seed vigour, RNA-based rather than DNA-based methods need to be employed.

The results also indicated that seed imbibition prior to heat treatment is crucial for both metabolome and metagenome composition, which can be expected since imbibition initiates the germination process and concomitantly provides suitable moisture and nutritional conditions for propagation^31,32^. This explains the lack of difference in metabolically decisive compounds in non-imbibed samples, but also the different changes in bacterial and fungal species diversity and richness compared with imbibed samples. Microorganisms colonising seeds differ in their ability to utilise organic compounds released during germination, leading to shifts in microbial community composition. However, the level of infection was similar in imbibed and non-imbibed seeds, indicating that the pathogens causing disease symptoms were not outcompeted by increasing species diversity and richness.

To conclude, myo-inositol: glucose ratio may be a suitable predictor of the impact of thermal treatment intensity on winter wheat seeds. However, to develop an accurate model for prediction, the window of treatment intensity needs to be narrowed to 50–70℃. DNA-based metagenomics analysis was unable to identify any key organisms, or groups of organisms, that can predict the impact of thermal treatment intensity.

## Electronic supplementary material

Below is the link to the electronic supplementary material.

Supplementary Material 1

## Data Availability

Analysed Data from the Illumina sequencing can be found in the supplementary information and are deposit at NCBI accession number: PRJNA1034801. Data from the metabolomics can be requested from the authors.
