# Supplementary material for: Impact of thermal seed treatment on spermosphere microbiome, metabolome and viability of winter wheat
Source: Sci Rep. 2024 Nov 8;14:27197. doi: 10.1038/s41598-024-78575-0 (PMC11549219; doi:10.1038/s41598-024-78575-0)
Supplement: Supplementary file 1 — Supplementary Material 1 [file 41598_2024_78575_MOESM1_ESM.docx]

## *Scientific Reports* Supporting Information

Article title: **Impact of thermal seed treatment on spermosphere microbiota, metabolome and viability of winter wheat**

Authors: Maria E. Karlsson^1*^, Gustaf Forsberg^2^, Anna Karin Rosberg^1^, Christian Thaning^2^, Beatrix Alsanius^1^

Table 1. Species specific primer used in this study to quantify *Fusarium* spp and *Microdochium* spp with ddPCR.

Figure S1. Relative abundance of (A) bacterial and (B) fungal phyla in milled winter wheat seed samples, grouped by heat intensity level and imbibition treatment.

Figure S2. Relative abundance of (A) bacterial and (B) fungal families in milled winter wheat seed samples, grouped by heat intensity level and imbibition treatment.

Figure S3. Relative abundance of (A) bacterial and (B) fungal genera in milled winter wheat seed samples, grouped by heat intensity level and imbibition treatment.

Figure S4. Relative abundance fungal species in milled winter wheat seed samples, grouped by heat intensity level and imbibition treatment.

Figure S5. Relative abundance of (A) bacterial and (B) fungal phyla on winter wheat seed coats, grouped by heat intensity level and imbibition treatment.

Figure S6. Relative abundance of (A) bacterial and (B) fungal families on winter wheat seed coats, grouped by heat intensity level and imbibition treatment.

Figure S7. Relative abundance of (A) bacterial and (B) fungal genera on winter wheat seed coats, grouped by heat intensity level and imbibition treatment.

Figure S8. Relative abundance of fungal species on winter wheat seed coats, grouped by heat intensity level and imbibition treatment.
